# Transforming children’s palliative care—from ideas to action: highlights from the first ICPCN conference on children’s palliative care

**DOI:** 10.3332/ecancer.2014.415

**Published:** 2014-03-31

**Authors:** J Downing, J Marston, MA Muckaden, S Boucher, M Cardoz, B Nkosi, B Steel, P Talawadekar, P Tilve

**Affiliations:** 1 International Children’s Palliative Care Network and Makerere University, PO Box 72518, Kampala, Uganda; 2 ICPCN, Bleomfontein 9301, South Africa; 3 Department of Palliative Medicine, Tata Memorial Centre, Mumbai, Maharashta 400012, India; 4 ICPCN, Durban 4000, South Africa; 5 Children’s Palliative Care Project, Indian Association of Palliative Care, Mumbai, Maharashta 400012, India; 6 ICPCN, Johannesburg 2000, South Africa

**Keywords:** children, declaration, education, integration, international, palliative care, research

## Abstract

The International Children’s Palliative Care Network (ICPCN) held its first international conference on children’s palliative care, in conjunction with Tata Memorial Centre, in Mumbai, India, from 10–12 February 2014. The theme of the conference, *Transforming children’s palliative care—from ideas to action*, reflected the vision of the ICPCN to live in a world where every child who needs it, can access palliative care, regardless of where they live. Key to this is action, to develop service provision and advocate for children’s palliative care. Three pre-conference workshops were held on 9 February, aimed at doctors, nurses, social workers, and volunteers, and focused around the principles of children’s palliative care, and in particular pain and symptom management. The conference brought together 235 participants representing 38 countries. Key themes identified throughout the conference included: the need for advocacy and leadership; for education and research, with great strides having been taken in the development of an evidence base for children’s palliative care, along with the provision of education; the importance of communication and attention to spirituality in children, and issues around clinical care, in particular for neonates. Delegates were continually challenged to transform children’s palliative care in their parts of the world and the conference culminated in the signing of the ICPCN Mumbai Declaration. The Declaration calls upon governments around the world to improve access to quality children’s palliative care services and made a call on the Belgian government not to pass a bill allowing children to be euthanised in that country. The conference highlighted many of the ongoing developments in children’s palliative care around the world, and as she closed the conference, Joan Marston (ICPCN CEO) challenged participants to take positive action and be the champions that the children need, thus transforming children’s palliative care.

## Introduction

The International Children’s Palliative Care Network held its first international conference on children’s palliative care, in conjunction with Tata Memorial Centre, Mumbai, India. The conference was held at Tata Memorial Centre from the 10–12 February 2014 with pre-conference workshops on 9 February 2014.

The World Health Organization (WHO) states that ‘*Palliative care for children represents a special, albeit closely related field to adult palliative care and is the active total care of the child’s body, mind and spirit, and also involves giving support to the family. It begins when illness is diagnosed, and continues regardless of whether or not a child receives treatment directed at the disease. Health providers must evaluate and alleviate a child’s physical, psychological, and social distress. Effective palliative care requires a broad multidisciplinary approach that includes the family and makes use of available community resources; it can be successfully implemented even if resources are limited. It can be provided in tertiary care facilities, in community health centres and even in children’s homes*’ [1].

The International Children’s Palliative Care Network (ICPCN) was established in 2005 in Seoul, South Korea, by a group of children’s palliative care practitioners from 15 countries who realised that by sharing knowledge, materials and experiences, and collaborating on advocacy, life-limited children in each country and region would benefit. The ICPCN is led by a team of recognised leaders working in children’s palliative care from across the world and from its initial beginning in 2005, it now has over 1500 members (individual and organisational) from more than 100 countries in all parts of the world. It is registered as a Charity in the United Kingdom and as a Public Benefit Organisation in South Africa.

The Declaration of Korea, upon which the ICPCN was founded, asked that ‘... *the voices of children and young people be heard, respected and acknowledged as part of the development of palliative care worldwide*’ [2]. Thus, the vision of the ICPCN is ‘to live in a world where children’s palliative care is acknowledged and respected as a unique service and every child and young person with life-limiting or life-threatening conditions, and their families, can receive the best quality of life and care regardless of which country they live in.’ ICPCN aims to:

1. Help services across the world to develop and meet the total care and support needs of life-limited children and their families.
2. Advocate and raise awareness of children’s palliative care and the specific needs of life-limited or life-threatened children and their families.
3. Develop a strong ICPCN membership from children’s palliative care services across the globe.
4. Facilitate communication and sharing of resources, information, and research worldwide, and to provide a ‘one stop shop’ for information relating to children’s palliative care.
5. Campaign for the global development of children’s palliative care services.
6. Enable the sharing of expertise and information between children’s palliative care practitioners.
7. Increase the international evidence base for children’s palliative care [3].

It was anticipated that this first ICPCN conference on children’s palliative care would promote these aims, enabling individuals and organisations from around the world to meet and learn from each other; raising awareness of children’s palliative care, specifically in India, strengthening the membership, facilitating communication and sharing, and demonstrating the increase in the international evidence base for children’s palliative care and, most importantly, advocating for the ongoing global development of children’s palliative care.

## Tata Memorial Centre

The conference was held in conjunction with the Tata Memorial Centre, one of India’s leading cancer hospitals, serving both government and private patients. Approximately 43,000 patients visit the clinics annually from all over India and neighbouring countries. Over 1000 patients attend the outpatients department daily for medical advice, comprehensive care, or for follow up treatment and around 70% are treated free of charge.

Alongside clinical care (with 6000 patients receiving radiotherapy and chemotherapy annually), clinical research programmes, randomised trials and education, and training programmes contribute to improved delivery of care and developing and maintaining high standards of care. Strategies are in place for early detection and diagnosis, treatment, rehabilitation, pain relief, and palliative care (including end-of-life care) such that a comprehensive and multidisciplinary approach to cancer care has been developed. As a recognised training centre by organisations such as the World Health Organization (WHO), International Atomic Energy Agency (IAEA), and the Union for International Cancer Control (UICC), the hospital is a post-graduate teaching centre and is affiliated to the Homi Bhabha National Institute (HBNI). Annually, there are approximately 450 undergoing training through the hospital, including about 80 post-graduate students undertaking Masters or Doctorate programmes [4].

In 1996, the palliative care unit opened and developed rapidly with the commencement of weekly clinics and since its inception, over 15,000 patients have been seen by the palliative care unit. In 1998, a medical registrar was appointed to start a home care service in the area, and this has now been expanded to also include nurses, volunteers, counsellors, and social support workers. Whilst initially focusing on the provision of care for adults, children’s palliative care services commenced in 2001. The team provides free medical, nursing, and psychosocial care for patients in their own home as well as emotional support and guidance to family members, along with bereavement support. Each year, approximately 1400 adults and 200 children are cared for by the palliative care unit in the hospital, with numbers consistently rising. The home service supports around 1100 families per year, providing holistic care for the patient and their family. Throughout the hospital, education and training in palliative care is a core component of the service, with training being provided to medical and nursing professionals along with volunteers and family members. They run a two year fellowship programme in palliative care and also provide training for the Indian Association of Palliative Care courses [5].

In October 2010, the Children’s Palliative Care Project in India was initiated in Maharashtra by the Indian Association of Palliative Care (IAPC). The project is funded by the UK Department for International Development (DFID) and is coordinated by the ICPCN and Help the Hospices (UK), with the Tata Memorial Centre providing mentorship. Three sites for children’s palliative care have been set up through the project. First, at the paediatric centre of excellence for HIV in association with Lokmanya Tilak Municipal General Hospital (Sion Hospital) in Mumbai. Second, at a rural site at Jawahar in association with the National Rural Health Mission (NRHM), and finally at the MGM Hospital, Kalamboli. The aim of the project is to develop a sustainable model of responding to the needs of children with life-limiting conditions.

### Pre-conference workshops

Three pre-conference workshops were held on Sunday, 9 February—one for doctors, one for nurses, and one for social workers and volunteers. 108 participants attended the pre-conference workshops (45 Doctors, 23 Nurses, 18 Social workers, and 22 other professionals) from 13 countries (Australia, Belgium, Canada, Germany, India, Indonesia, Italy, Kenya, Myanmar, Nigeria, Sweden, Thailand, and the United States). The pre-conference workshop for doctors focused on pain and symptom management and was chaired by Prof Julia Downing (ICPCN), Dr Nimain Mohanty (India), and Prof Purna Kurkure (India). The workshop aimed to give participants the opportunity to learn about pain and symptom management in children’s palliative care from international experts who were speaking at the conference. Alongside issues around pain and symptom management the workshop also explored the principles and practices of palliative care in children and the importance of good pain and symptom assessment and management, along with communication in children’s palliative care. International experts facilitating on the workshop included Dr Delia Birtar (Romania), Prof Mary Ann Muckaden (India), John Collins (Australia), Dr Chantal Wood (France), Dr Ross Drake (New Zealand), Dr Jan Aldridge (United Kingdom), and Prof Julia Downing (ICPCN). The importance of pain and symptom control and the principles underpinning effective management were discussed. The challenges of complex pain and symptoms were explored utilising various case studies, and exemplars from different situations, culminating in a case study that was discussed by the group and brought together the different topics that had been shared throughout the day. 100% of the participants who completed the evaluation form rated the course as good or very good (on a five-point Likert scale) in terms of organisation, usefulness to their own work situation and their overall assessment of the workshop. Participants appreciated the opportunity to discuss cases and have ‘*free and frank discussions with professionals drawn from various strata world-wide*.’ The session on communication, held in the afternoon, was the session that the majority of participants found most helpful, with communication being one of the topics recommended for future workshops. One participant commented that ‘*this pre-conference workshop has given me new ideas about palliative care*,’ *another that ‘experts from different countries, each with a unique viewpoint, made for a very stimulating experience*.’

The workshop on Principles and Practices of children’s palliative care for nurses, was chaired by Prof Susan Fowler-Kerry from Canada and Assoc Prof Caprice Knapp from the United States. Joan Marston, Chief Executive of the ICPCN, opened the workshop by introducing participants to children’s palliative care, discussing the principles and practices, along with global and national development. She challenged participants, stating that children with palliative care needs are often too young, too sick, or too disabled, and parents are too tired and need their energy to care for their child and their family, so if we do not champion these children, who will? Topics covered included the puzzle of pain assessment (Prof Susan Fowler-Kerry, Canada), understanding how pain medications work (Dr Ross Drake, New Zealand), the art of communicating (Dr Jan Aldridge, UK), spirituality (Natasha Pederson, Norway), the need for advocacy (Busi Nkosi, ICPCN), and a case for quality improvement in practice (Assoc Prof Caprice Knapp, USA). 95% of the participants who completed the evaluation form rated the course as good or very good (on a five-point Likert scale) in terms of organisation, usefulness to their own work situation and their overall assessment of the workshop. The interactive nature of the workshop was appreciated and different people found different sessions most useful and appropriate to their workplace. Nurses were challenged throughout the day to develop knowledge and skills in children’s palliative care, to integrate palliative care principles into their practice, to support parents, ensure adequate pain and symptom control, including advocating for opioids to treat pain, and importantly, to become champions for children’s palliative care as ‘*We must be the change we wish to see in the world*’ (Mahatma Gandhi).

The third of the pre-conference workshops was on psychosocial issues and communication in children’s palliative care and was aimed at social workers, psychologists, and volunteers. It was chaired by Dr Mrunal Marathe, with all of the facilitators coming from India—Dr Jayita Deodhar, Savita Goswami, Dr Sunil Dhilwal, Dr Seema Rao, and Dr Murnal Marathe. The programme was divided into three parts starting with a session on communicating with children. Following this interactive session, issues pertinent to care of the caregivers were discussed, and finally the place of care, and how this impacts the services provided, and the model of service provision. The interactive nature of the workshop was appreciated by participants, and the majority of participants felt that the workshop was appropriate to their practice and anticipated that their practice would change as a result of the workshop.

## Background to the conference

Palliative care for children represents a special, albeit closely related, field to adult palliative care. However, in many parts of the world, many of the children who need palliative care cannot access it, thus the provision of high-quality palliative care for children is a global concern [6]. Whilst over recent years, there have been rapid advances in the development of palliative care [7], with the commitment to pain relief being a human right [8], and palliative care being integrated into national health policies/strategies, there is still inadequate access to palliative care for children worldwide [9]. This conference provided a platform for sharing and demonstrating significant advances in the provision of children’s palliative care throughout the world.

A systematic review of children’s palliative care provision around the world, undertaken in 2011, found that 65.6% of countries had no known children’s palliative care activities, 18.8% had capacity building activities, 9.9% localised provision, and only 5.7% of countries had children’s palliative care provision that was reaching mainstream providers [10]. More recently, the newly published Global Atlas of Palliative Care at the End of Life [9], estimated that 6% of the global need for palliative care (based on mortality figures) is in children (<15 years of age), i.e., about 1.2 million children or 63 children out of every 100,000 will require palliative care at the end of life. 49% of these children are from the African region, followed by 24% from Southeast Asia, and 12% from the Eastern Mediterranean regions. The majority of children needing palliative care at the end of life have non-malignant diseases (ranging from 78% in the African Region to 91% in the Eastern Mediterranean Region), with the Western Pacific and European regions having the highest percentage of children with cancer in need of palliative care at the end of life (14.4% and 12.7%, respectively). This is important as it is predicted that the number of cancer patients in developing countries will increase significantly in the future.

A study carried out by UNICEF and the ICPCN recently looked at the need for children’s palliative care in Kenya, South Africa, and Zimbabwe [11]. Building on from the work of the Atlas, along with work by Fraser *et al* [12], this study looked at need based mainly on prevalence data. Fraser *et al* [12] estimated that in England the population prevalence for children needing specialised palliative care is 32 per 10,000 children. However, due to the higher incidence of HIV/AIDS, along with a lack of access to well-functioning health care systems, it was anticipated that the prevalence would be a lot higher in these three sub-Saharan African countries. Indeed, the study found that the rate per 10,000 children ranged from 120.05 in Kenya to 180.63 in Zimbabwe, with the number of children benefiting from palliative care services in the three countries being less than 5% of need in South Africa and Zimbabwe, and less than 1% of need in Kenya.

Thus, the need for ongoing development of children’s palliative care throughout the world is great. However, through ongoing collaboration and advocacy, strides have been made to advance children’s palliative care globally, not just in terms of service provision, but also in terms of access to services, and to the essential medicines for palliative care provision. This conference provided a platform for individuals new to children’s palliative care to hear what is going on around the world, to learn from other programmes, and also those experienced in the field, to share their experience, and to continue to learn and develop. It provided an opportunity to share together our vision of transforming children’s palliative care; putting ideas into action; hearing of successful research, education, programmes, and projects—all aimed at improving the care of children and families facing life-limiting and life-threatening conditions.

## Conference summary

The theme of the conference was *Transforming children’s palliative care—from ideas to action*, and was based around different themes including: (1) Action in clinical care; (2) Action in Programme Development; (3) Action in Education; (4) Action in Research; (5) Action in Advocacy; and (6) Action in Leadership. The scientific programme included a variety of plenary sessions (eight papers), 73 oral breakout presentations, including workshops, and 43 poster presentations. Oral and poster abstracts were accepted from 30 countries, with institutional representation spanning organisations that have spearheaded the development of children’s palliative care, alongside institutions that are just beginning to integrate children’s palliative care, thus demonstrating the breadth of delegates represented. Applied workshops, which aimed to apply what is being discussed to our own situations included a mixture of presentations and interactive discussion and covered the areas of advocacy, leadership, pain, research, communication, and spirituality and education.

The conference, held at the Tata Memorial Centre in Mumbai, India, brought together 235 delegates representing 38 countries from around the world. Delegates attended from: Argentina, Australia, Bangladesh, Belarus, Belgium, Cambodia, Canada, China, France, Germany, Ireland, India, Indonesia, Ireland, Italy, Japan, Kenya, Kuwait, Malaysia, Malawi, Myanmar, the Netherlands, New Zealand, Nigeria, Norway, Romania, Russia, Serbia, South Africa, Swaziland, Sweden, Tajikistan, Thailand, Uganda, the United Kingdom, the United States of America, Zambia, and Zimbabwe. The conference brought together clinicians, academics, advocates, clergy, researchers, social workers, managers, policy makers, Ministry of Health officials, members of the press, and national palliative care associations.

Delegates were given a traditional, warm welcome by their Indian hosts. The conference was opened by Barbara Gelb and Prof Mary Ann Muckaden, Co-Chairs of the ICPCN, Joan Marston, the Chief Executive, and Prof Julia Downing, the Scientific Chair of the conference. Before the lighting of the traditional lamp, a young boy sang a traditional song calling the blessing of the gods upon the conference. The opening address was given by Sister Frances Dominica, where she shared her inspiring story of opening the first children’s hospice, Helen House, in Oxford in the United Kingdom. In her talk, she spoke of the need for those who provide care to ‘walk the walk’ alongside the children and their families and to learn the skills that the families use to care for their child, suggesting adaptions where necessary. Carers are needed who are courageous enough to remain alongside the family ‘into that place of unknowing.’ She summarised children’s palliative care as being ‘*about living life to the full as long as that is possible; to make the most of what life there is and to always keep the family in the very centre of the care provided*.’ [13].

Assoc. Prof John Collins, Head of Service at the Department of Pain Medicine and Palliative Care Service at the Children’s Hospital at Westmead (New South Wales, Australia) then gave an insightful overview of the educational needs in Paediatric Palliative Care today. Drawing on the wisdom of Gandhi, he spoke of the need for a basic education in children’s palliative care for all who work with children needing palliative care, and reminded delegates that knowledge and work are not separate, that education is lifelong and holistic. Different people working in children’s palliative care need different levels of education, those who learn should be judicious about the available resources, and collaboration and sharing are vital to the development of the specialty as is evidence based research. He borrowed the closing words of his talk from Nelson Mandela who said ‘*Education is the most powerful weapon which you can use to change the world*’ [13].

The Inaugural Ceremony was held in the evening of the first day, following a series of inspiring breakout sessions and applied workshops. In his address, Mr Suresh Shetty, Minister of Health and Family Welfare, Government of Mahatashtra, stated his support for a further roll out of children’s palliative care throughout the state of Maharashtra, and recognised the work done by the children’s palliative care team based at Tata Memorial Centre. He was joined in welcoming delegates by Dr RA Badwe, Director of the Tata Memorial Centre, and Dr AK D’Cruz, Head of Surgical Oncology at the Hospital. Following the ceremonial lighting of the lamp and speeches by the dignitaries present, delegates were treated to a display of traditional dancing from a group of colourfully clad children, many of whom are patients in the children’s palliative care programme. Reflecting on the success of the first day and the remarks by the Health Minister, Joan Marston had this to say, ‘*We were delighted to hear that the minister supports and recognises the need for palliative care for children in the entire Maharashtra state. This level of support is a testament to the dedication and success of the CPC Project team under the leadership of Dr Pradnya Talawadekar*’ [14].

Dr Stephen Liben, Associate Professor of Paediatrics in the Faculty of Medicine of McGill University and Medical Director of The Montreal Children’s Hospital Paediatric Palliative Care Programme in Canada, started the second day of the conference by looking at the issue of mindfulness and caring for the caregiver. He shared the North American idea that the only model for living is a consumer one—we want something, we shop for it and we buy it. Patients (those who suffer) then become transformed into ‘clients’ and then into ‘consumers.’ He then posed the questions: ‘What does that make clinicians? Are we service providers or caregivers? However, we cannot always buy health and in children’s palliative care we see people faced with the ultimate futility—that even money cannot always keep the most precious from being taken away... their children... He went on to discuss the issue of caregiver burnout and the fact that although we might not feel, as if we are in control, we *can* control our response to what is happening around us. Thus, the need for mindfulness—mindful-awareness—it is not easy, but we need to consider intention, attention, and attitude, and, above all, to practice being mindful.

Delegates were then apprised of the need to transform practice through research by Prof Myra Bluebond-Langner (Professor and True Colours Chair in Palliative Care for Children and Young People at University College London, Institute of Child Health, United Kingdom). She shared her journey through research as an anthropologist, studying the world of dying children, their siblings, and families. She discussed the importance of using research to transform practice saying that we need to utilise the findings of research to bring about change in practice, such that we can transform the care that we are providing to children and their families. However, in an international context, we need to be always mindful of different cultural and contextual factors.

Challenging ethical issues in children’s palliative care was the focus of the plenary session by Dr Richard Hain (Consultant and Lead Clinician in Paediatric Palliative Medicine in Wales) and Prof Mary Ann Muckaden (Professor and Head of the Department of Palliative Medicine at the Tata Memorial Centre). Dr Hain commenced the session by posing the question—Do we need ethics? He noted that there are a variety of helpful insights into medical ethics; however, none of them represents a coherent account of ethics in children. He said that for us working within children’s palliative care, it is not just ‘intention’ and ‘consequence’ that are important, but the action as well—we need to be the *right sort* of carers, making the *right decisions* for our patients, and for the *right reasons*. He ended with the thought that ethics is important in children’s palliative care, and depends on relationship as well as reason, and therefore, it makes personal demands on us as carers. Delegates were then introduced to the cultural context within India by Prof Muckaden, who went on to share three different case studies from her practice, each of which raised different ethical issues. Delegates had the opportunity to discuss the cases with each other, prior to Prof Muckaden sharing what actually happened in each situation.

Following a further afternoon of workshops and discussions, delegates were invited to attend the gala dinner with a theme of a traditional wedding feast, which included the painting of intricate henna designs on the hands of the women guests and the wearing of turbans for the men, as well as a display of a blend of contemporary and traditional dancing and singing. During the festivities, the award winning Bollywood actor, Kunal Kapoor, arrived as a surprise guest and was invited to light the traditional lamp. In his response to being thanked for his presence he commented, ‘I couldn’t think of anything more important than to give comfort and support to these children and their families.’ It is hoped that Kunal Kapoor will continue to provide support to help raise the profile of children’s palliative care within India [15].

The final day of the conference started by addressing the issue of neonatal palliative care—an area that has caused much debate and discussion. Dr Rut Kiman (Head of the Paediatric Palliative Care Team, Maternal-Infant Department at Professor A. Posadas Hospital—Argentina; and Senior Lecturer, Paediatric Department, School of Medicine, University of Buenos Aires) gave an overview of palliative care in the neonatal period in order to enhance care of babies with no curative options. She shared her experiences in Argentina through the discussion of various children for whom she had provided care and support. She also noted that around the world most neonatal deaths occur in hospital settings, generally few parents are allowed to take their babies home to die with appropriate support, or to a children’s hospice, where one is available. As the theme of the conference was about ‘ideas to action’—she challenged delegates to do what they can to make a difference in the provision of neonatal palliative care in their respective settings.

One of the greatest challenges noted by Dr Kiman was access to appropriate medications. This theme was developed further by Dr Jim Cleary, Associate Professor of Medicine, Medical Oncology Section; Director of Palliative Medicine at University of Wisconsin Hospital and Clinics; and Academic Medical Director of Hospice Care Inc. (USA). In his presentation, Dr Cleary discussed the global lack of access to opioids in children’s palliative care, noting that most low and middle income countries have significant regulatory barriers to the use of opioids and that there is a need for the global children’s palliative care community to collaborate and try and improve the situation throughout the world. In doing this, he also called upon those of us within children’s palliative care to utilise all forms of social media available as effective advocacy and communication tools.

Throughout the plenary sessions of the conference, delegates were treated to a selection of previews of the ‘Little Stars’ (www.littlestars.tv) series of short films, expertly filmed and directed by Mike and Sue Hill of Moonshine Movies (the makers of the Life Before Death films http://www.lifebeforedeath.com). These moving short films featured stories of children and young adults and their families benefitting from palliative care and had been filmed in South Africa, Australia, and the United States. Before the closing ceremony of the conference, Mike Hill explained the motivation behind making these films, and their plans to make many more, including a feature length film. Throughout the conference, delegates commented positively on the insightfulness of the films and the message that they told.

As the conference moved to a close delegates were given the challenge to transform children’s palliative care in their parts of the world and to move from ideas into action. Two such ways that Joan Marston encouraged them to do this included their support for the development of a children’s hospice in Mumbai and to sign the ICPCN Mumbai Declaration 2014. Joan shared with delegates the vision of two young entrepreneurs, Abishek Tatiya and Mansi Shah, who are determined to open the first Children’s Hospice in India to be known as ‘Happy Feet Home.’ Following a short film about the hospice, delegates were called on to assist where they could to help get this project off the ground.

Joan Marston then called upon delegates to sign a declaration which was issued at the end of the conference, to be known as the ICPCN Mumbai Declaration 2014. The declaration calls upon governments around the world to improve access to high-quality children’s palliative care services and it made a particular call upon the Belgian government not to pass a bill allowing children to be euthanised in that country.

**ICPCN Mumbai Declaration 2014**

We believe that all children (neonates, children, and young people) have the right to the best quality of life. When they have life-limiting conditions they have the right to high-quality palliative care to meet their needs.

We believe that euthanasia is not part of children’s palliative care and is not an alternative to palliative care. It is imperative that we work together to improve access to children’s palliative care around the world, including ensuring access to appropriate pain and symptom control.

We call on all governments to transform children’s lives through the development of children’s palliative care, and in particular we urge the Belgian government to reconsider their recent decision to allow euthanasia of children.

This includes:

1. Access to children’s palliative care within the children’s health care system.
2. Access to appropriate pain and symptom management (including medications) for all children.
3. Supporting children and their families to be able to live their lives to the best of their ability for as long as possible.

## Applied workshops

### Applied workshop on advocacy

An applied workshop was held on advocacy to encourage delegates to develop their advocacy skills, to show how they can make a difference, and to help them to develop an advocacy plan for their country/ region. ICPCN’s Advocacy Officer Busi Nkosi, set the scene by discussing access to palliative care as the right of every child. This was followed by examples from the children’s palliative care project in India, where advocacy efforts have brought about change in the laws enabling access to pain medication and a resolution by the Government of Maharashtra to implement children’s palliative care. Lessons from advocating for the integration of palliative care by traditional healers in South Africa were discussed, leading to the development of a training manual for traditional healers with training rolled out by the National Department of Traditional Medicine. The ICPCN’s advocacy strategy was discussed and delegates were then taken through eight steps to good advocacy by Dr Stephen Connor (USA) and Sharon Baxter (Canada), how to use advocacy data and reports, such as the Global Atlas [9] and the WPCA advocacy toolkit [16]. Delegates then worked together in small groups to identify goals for children’s palliative care advocacy in different parts of the world, such that they could move from ‘ideas to action.’

### Applied workshop on leadership

Health care teams function best when leaders are strong and there is alignment between leaders and their colleagues thus an applied workshop was held to cover issues of leadership, and to try and develop leaders within the field. The first part of the workshop focused on self-assessment and skill development in communication and generating alignment and was led by Assoc Prof Caprice Knapp (USA) through a series of presentations and interactive activities. These themes were then developed further by Andre Wagner (South Africa) and Barbara Gelb (UK) who addressed issues of diversity, organisational development, engaging individuals in debate around children’s palliative care and a discussion of some of the debates, dilemmas, and opportunities within children’s palliative care in the UK. It also looked at how we as leaders in the field can take advantage of these. Delegates were called upon to stimulate debate at every level in order to prepare for the future and lead the development of children’s palliative care into the future.

### Applied workshop on research

The ongoing development of an evidence base for children’s palliative care is a theme that has been discussed on many occasions, with a review of palliative care for children in sub-Saharan Africa demonstrating the lack of evidence published on the work being done [17]. Thus, it was exciting to hold an applied workshop where the results of new and important research within the field could be discussed. ICPCN’s Education and Research Consultant, Prof Julia Downing, opened the workshop by discussing the results of a Delphi study conducted to identify global priorities in research for children’s palliative care. Many of the delegates present had participated in the study and were keen to hear the results. Within the top ten priorities for research, estimating the need for children’s palliative care was identified as a priority. Both of the following studies addressed this area with Dr Richard Hain (UK) discussing the development of a Directory of Life-limiting conditions and how it can be used, along with its limitations. This was followed by a presentation of the results of a study conducted by UNICEF and ICPCN on the development of a methodology for estimating the need for palliative care, sharing results from Kenya, South Africa, and Zimbabwe [11]. The presentations evoked much discussion and delegates were keen to ask questions and discuss the next steps. There was a feeling of anticipation within the workshop with delegates looking forward to hearing about future research studies and findings and being involved where appropriate.

### Applied workshop on pain

The issue of assessment and management of pain in children is an area that causes much concern and debate therefore one of the applied workshops focused on this area. Topics ranged from the role of nurses in the management of pain, common pain syndromes in children with acute Leukaemias, the use of opioids in children, and how to improve pain control in children around the world. Interactive sessions included the use of Graded Motor Imagery for complex pain (Michael Sangster, Canada) and the use of hypnosis for pain and palliative care (Dr Chantal Wood, France) thus ensuring a focus on both pharmacological and non-pharmacological methods of pain management. The workshop was well attended, with delegates appreciating the opportunity to discuss issues around pain management in children.

### Applied workshop on communication and spirituality

This practical workshop, run by Dr Mrunal Marathe (India) explored some of the key aspects of communication required in children’s palliative care, with the goal of communication being to understand and acknowledge the concerns and needs of children, and to support them through the range of emotions that they will experience. Involving the child in decision-making was also discussed along with a range of methods to help do this. Likewise, the session on spirituality, conducted by Dr Mamta Manglani, Dr Ratna Sharma, and Dr Pradnya Talawadekar, discussed the meaning of spirituality, clarifying the differences between religion and spirituality, and the spiritual support that can be provided within children’s palliative care. This workshop drew upon experiences from within Mumbai and provided delegates with an opportunity to explore some of these issues within the Indian context.

### Applied workshop on education

Education is one of the pillars within the public health approach to palliative care development [18]. Thus, the workshop explored issues around how we can enrich our programmes through education (Jody Chrastek, USA), how we can learn from evaluation of our training programmes (Marie Friedel-Castorini, Belgium), and how we can utilise competencies for children’s palliative care. The European Association of Palliative Care has recently published some core competencies for children’s palliative care education [19], and these were shared with participants, along with the process of development and some of the resources available. Delegates were encouraged to read these competencies and utilise them as appropriate in their educational practice.

### Breakout sessions

Alongside the applied workshops, numerous breakout sessions were held. These sessions covered a variety of topics including clinical care, programme development, transitions, research, neonatal palliative care, and arts based therapies. Sessions were described as enriching, informative, exhilarating, and challenging, and throughout these presentations gave rise to much debate and discussion.

## Conclusion

The last few years have seen significant developments in children’s palliative care globally. This first ICPCN conference on children’s palliative care highlighted many of these developments and served as a unique opportunity to bring delegates from multi-disciplinary backgrounds together to serve as a lynchpin for children’s palliative care provision and development throughout the world. The impressive and substantial line up of speakers for the plenary sessions added academic weight to the conference and their presentations covered a broad range of topics. The standard of the presentations and the posters was also exceptionally high and a testament to the excellent selection process carried out by the conference scientific committee. The pre-conference workshops also proved to be popular with over 100 registrations on the day.

As she reflected on the enormous success of the conference, ICPCN Chief Executive Joan Marston commented ‘*Taking the decision to hold our very first international conference was an act of courage, but deciding to hold it in India, where a conference of this nature had never been held before, was a giant leap of faith. And it paid off... We wanted to bring the foremost experts in the field to a country where there is an overwhelming need for children’s palliative care services and expertise and we were successful in doing just that*’ [20]. Thus, at the close of the conference she left the delegates with the final challenge to ‘*take positive action and be the champions that these children need, thus transforming children’s palliative care by turning their ideas into action*.’

## Conflict of Interest

The authors declare that they have no conflict of interest.
